# Preparation and Flame-Retardant Mechanism of MgAlZn-Based Hydrotalcite-like Coal Spontaneous Combustion Inhibitor

**DOI:** 10.3390/ma18010070

**Published:** 2024-12-27

**Authors:** Lei Li, Yaohui Li, Zulin Li, Lingling Wu, Jingchuan Gou, Xingrong He, Chenxi Xu, Caijing Xie, Wanyue Wu

**Affiliations:** 1 School of Chemical Engineering, Sichuan University of Science and Engineering, Zigong 643000, China; lilei20150620@yeah.net (L.L.); 18161106317@163.com (Z.L.); 15328286851@139.com (L.W.); 15760592990@139.com (J.G.); 15729854247@163.com (X.H.); xcx18980796495@163.com (C.X.); xcj030207@163.com (C.X.); 2 Shanxi GaopingNanHe Coal Industry Co., Ltd., Jincheng 048000, China; 3 School of Management, Sichuan University of Science and Engineering, Zigong 643000, China; gp230010@student.uthm.edu.my

**Keywords:** MgAlZn-LDHs, flame retardant, coal spontaneous combustion

## Abstract

In this work, the coprecipitation approach was successfully used to create Mg-Al hydrotalcite-like inhibitors modified with varying amounts of Zn, and their characteristics were assessed. The findings indicate that the flame retardancy of Mg-Al hydrotalcite (MgAl-LDHs) is not significantly affected by Zn content. By adding MgAl-LDHs, the temperature at which the exothermic reaction started to occur was raised from 146.2 °C to 193.6 °C, according to the test of spontaneous combustion tendency. In the excavation route, the utility model can serve as a temporary fire prevention and extinguishment tool. Furthermore, the analysis of the functional group changes during the reaction was conducted using FTIR. After applying MgAl-LDHs, the oxidation of organic groups on the coal surface was clearly prevented, indicating that the inhibitor had a substantial flame-retardant effect on coal. In conclusion, this work creates a material that resembles hydrotalcite and is easy to use, inexpensive, and effective in preventing coal from spontaneously combusting.

## 1. Introduction

China has few energy resources, including oil and natural gas, but its coal reserves are the greatest in the world and are also the most essential and commonly used kind of energy [1]. The primary energy source for industrial production is coal, but during storage or transportation, the oxidation of surface groups in the coal produces heat. In addition to releasing a lot of harmful gasses such as carbon monoxide, carbon dioxide, nitrogen, and sulfur oxides, the spontaneous combustion of coal would result in resource loss [2]. It is simple to have a negative impact on the natural environment, locals’ daily lives, and personal safety. As a result, both domestic and foreign academics are very interested in the prevention and management of coal spontaneous combustion brought on by fire.

Coal spontaneous combustion is a physically chemical process that is automatically accelerated and nonlinear. It involves turbulent flow, heat and mass transfer, phase transition, and complex chemical reactions [3]. A great deal of research has been done on coal spontaneous combustion by many researchers. Some of the primary theories that have been proposed are the coal-oxygen complex theory [4], the pyrite cause theory, the bacteria cause theory, and the phenol gene cause hypothesis [5]. Among these, the coal-oxygen interaction theory has gained widespread acceptance among academics both domestically and internationally due to its validation in both laboratory and practical settings. The hypothesis of the coal-oxygen combination states that heat and coal-oxygen combination are the primary causes of coal spontaneous combustion. There are a lot of active functional groups in coal, and these functional groups behave differently during oxidation reactions [6]. In actuality, the temperature of the coal will rise and the oxidation reaction will speed up if the heat produced by the oxidation of coal is greater than the heat lost by convection between coal and air. This will release more heat and make the temperature of the coal easier to reach the ignition point and spontaneous combustion. Thus, the primary factor influencing coal’s spontaneous combustion during oxidation is the exothermic impact of coal.

There are currently few reports on the process of active group change in coal during oxidation and its effects on the exothermic characteristics of coal, primarily related to macro−qualitative research, for the study of coal oxidation heat release characteristics of the thermodynamic calculation methods [7]. Zhang’s programmed temperature−rise experimental rig was used to numerically simulate the oxidation process of active groups based on the properties of the infrared spectrum of coal at various temperatures. This research will serve as the foundation for a more methodical study of the properties of the coal oxidation reaction using cutting−edge technology. By controlling the angle at which the active groups in the coal participate in the oxidation reaction and by absorbing the exothermic heat of the coal oxidation reaction, this approach will effectively control the spontaneous combustion of coal by lowering the rate of oxidation and heat release from the coal.

The inhibition theory of coal spontaneous combustion is found to be associated with the mechanism of coal oxidation [8]. The inhibitors cooperate through a variety of mechanisms, including heat absorption, moisture and temperature retention, oxygen isolation, and absorption, covering the active center, chain reaction inhibition or interruption, asphyxiation of inert gases, and the inhibition of coal’s ability to self−heat and self−ignition. Utilizing inhibitors, a novel technology has been developed to prevent and control coal mine fires. Several coal miners and associated academics in industrialized nations have conducted several trials and uses of this technology. Currently, physical, chemical, and novel composite inhibitors are the three types of inhibitors that are most frequently utilized. Every type of inhibitor has a restricted spectrum of application [9].

A type of inorganic substance with a layer structure that resembles hydrotalcite and has a lot of anions can participate in an ion exchange reaction in an anionic aqueous solution [10]. The general formula for this reaction is [M^2+^_1−x_M^3+^_x_(OH)_2_]^q+^(X^n−^)_q/n_•yH_2_O]. To create bimetallic oxides, a large number of carbonates or other transparent ions that filled the interlayer were calcined at a high temperature and then dried. The hydrotalcite-like compounds that were calcined were placed in an anionic solution. The bimetallic oxides exhibit a memory function, allowing anions from the solution to fill the interlayer and restore the original ordered layer structure. Compounds that resemble hydrotalcite are very thermally stable. OH^−^ and CO_3_^2−^ separate water and CO_2_ at high temperatures, which can lower gas concentrations and separate oxygen during burning. This multi-stage thermal breakdown endothermic action also lowers coal’s heat absorption during heating. Heat absorption is high, condensed phase forms on the polymer surface, diffusion of the combustion surface is decreased, and the high temperature of combustion is decreased during the flame retarding process [11].

In this work, the experimental platform was used to prepare the Mg-Al hydrotalcite-like compounds modified with varying amounts of Zn. A substance that resembles hydrotalcite but is safe for workers and does not corrode machinery is created. Low cost and simplicity characterize the synthetic method.

## 2. Experiment and Method

### 2.1. Chemicals and Reagents

Aluminum chloride (AlCl_3_, CAS 7446-70-0), zinc chloride (ZnCl_2_, CAS 7646-85-7), magnesium chloride (MgCl_2_, CAS 7791-18-6), sodium hydroxide (NaOH, CAS 1310-73-2), sodium carbonate (Na_2_CO_3_, CAS 497-19-8), and calcium chloride (CaCl_2_, CAS 10043-52-4) were purchased from Chengdu Kelong reagent company, Chengdu, China.

### 2.2. Sample Preparation

#### 2.2.1. Preparation of Hydrotalcite-like Slurry

To create solution A, a metal salt solution, 20.258 g MgCl_2_, and 12.075 g AlCl_3_, were first mixed in 180 mL of deionized water and thoroughly agitated. In order to make Solution B, 80 g NaOH and 53 g Na_2_CO_3_ were combined with 1 L of deionized water. Solution B was gradually added to solution A using a titrating funnel while stirring until the pH reached roughly 10. To create a slurry that resembled hydrotalcite, the slurry was churned for an additional hour after the pH value of the reaction solution had stabilized [12].

#### 2.2.2. Preparation of Hydrotalcite-like Solid Powder

After crystallizing in a water bath at 65 °C for 17 h, the hydrotalcite-like slurry was centrifuged and cleaned with deionized water until it was Cl^−^ free and had a pH of 7. To create a product that resembles hydrotalcite, the slurry was dried for 24 h at 65 °C in an oven, and the dry solid was then ground into powder.

#### 2.2.3. Proportion Screening of Metal Zn Elements

The hydrotalcite-like compounds modified by Zn element in varying quantities were generated in order to examine the flame retardancy of hydrotalcite-like compounds formed by adding Zn element to coal. The preparation procedure essentially follows the content mentioned above. The addition of 12.65 g (n(Mg)/n(Zn) = 1) or 25.30 g (n(Mg)/n(Zn) = 2) of ZnCl_2_ to the metal salt solution A was the only variation.

### 2.3. Experiment Procedure

#### 2.3.1. Collection and Preparation of Coal Samples

In accordance with GB/T482~2008 [13], fresh coal samples were gathered from Qiyi coal industry’s No. 9 coal seam and sent to the laboratory wrapped in fresh−keeping film. The middle portion of the samples was taken for shrinking, crushing, and screening, resulting in 100–200 mesh-size samples that were placed in sample bags, labeled, and ready for use. Detailed process in Figure S1 of Supplementary Materials.

#### 2.3.2. Measurement of Resistivity

The coal resistance test platform was filled with 8 g of samples at a rate of 100 mL·min^−1^. The temperature was programmed to be between 30 and 300 °C, with a heating rate of 5 °C per minute. The exhaust gas was collected using an air bag at a fixed temperature of 150, 200, 240, 270, or 300 °C. Gas chromatography was used to identify CO-concentration. Of them, one sample uses fresh coal, and the detection result is the CO concentration of the naked coal; the other sample uses coal that has had the inhibitor added to it, and the sample CO concentration is the result of the detection. The inhibitor-added samples were produced via straightforward physical mixing, or the mechanical blending of hydrotalcite-like substances with new coal powder in accordance with a predetermined mass ratio. Here’s how to determine resistance rate:

(1) Resistance rate%=C(CO)bare coal−C(CO)SampleC(CO)bare coal.

In this equation, C(CO) represents the concentration of CO.

#### 2.3.3. Characterization

The morphology of the samples was observed by cold field emission scanning electron microscopy. (SEM, Hitachi S4800, Yokohama, Japan). The infrared signals of the surface groups of hydrotalcite-like compounds were detected by Fourier transform infrared spectroscopy (FTIR, Bruker V70, Munich, Germany). The thermogravimetric curves of hydrotalcite-like samples during heating were measured by a synchronous thermal analyzer (TG-DSC, Netzsch STA449F3 Germany). See Supplementary Materials Table S1 for details.

#### 2.3.4. Determination of Spontaneous Combustion Tendency of Coal

The following is the test procedure for coal’s propensity for spontaneous combustion: 40 g of fresh coal sample should be ground to a 32–80 mesh size and placed in a special sample tank for the coal’s spontaneous combustion tendency platform. The temperature of the coal sample and the furnace’s temperature are recorded, respectively, and the two temperature curves are continuously monitored until the point at which the two temperature lines intersect the spontaneous combustion of the coal sample is reached. By comparing the two temperature intersections of coal samples treated with pure coal and inhibitor, the flame-retardant effect of hydrotalcite-like compounds can be assessed. 

## 3. Results and Discussion

### 3.1. Characterization of Hydrotalcite-like Compounds

**Figure 1:**
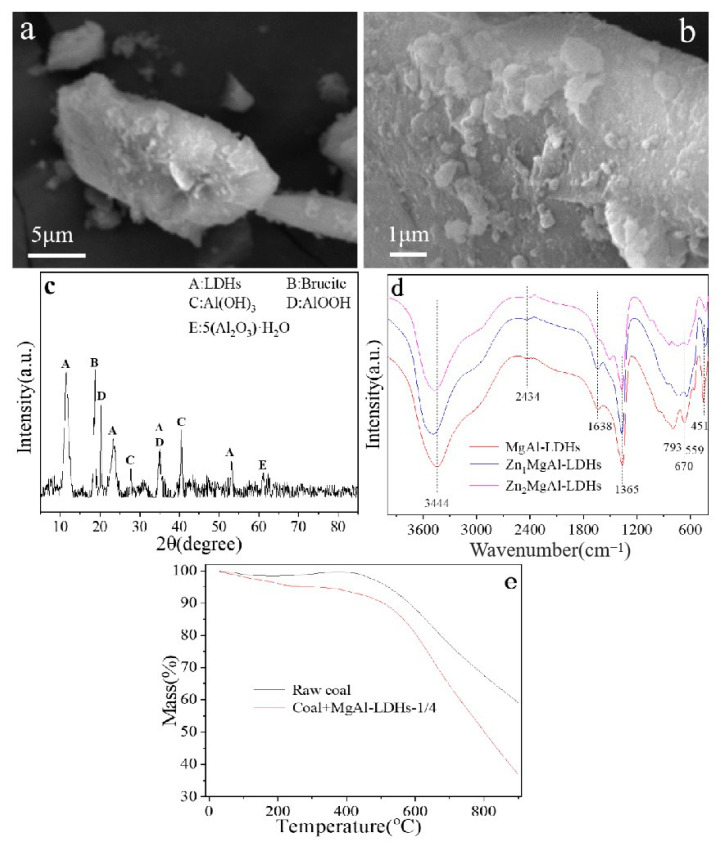
(**a**,**b**) Scanning electron microscopy of hydrotalcite-like compounds. (**c**) X−ray diffraction of hydrotalcite-like compounds. (**d**) FTIR spectra of hydrotalcite-like compounds with different Zn contents. (**e**) Thermogravimetric curves of raw coal, coal, and MgAl-LDHs mixtures.

SEM was used to examine the morphology of compounds that resembled hydrotalcite (Figure 1a,b). The figure makes it clear that the MgAl-LDHs have a lamellar morphology. The consistent distribution of lamellar size provides a clear view of the lamellar structure. XRD was used to examine the produced hydrotalcite-like compounds (Figure 1c). The distinctive peaks of Al(OH)_3_, AlOOH, brucite, and LDHs are easily visible. When paired with SEM and XRD, the synthesized sample exhibits a characteristic hydrotalcite layered structure, high crystallinity, and uniform dispersion.

The FTIR spectra of hydrotalcite-like compounds with varying Zn concentrations in the 400–4000 cm^−1^ range are displayed in Figure 1d. The figure shows that MgAl-LDHs have nine infrared absorption peaks at 451, 559, 670, 793, 1365, 1638, 2434, and 3444 cm^−1^. These peaks are consistent with the infrared signals that are typically observed on the surface of compounds that resemble hydrotalcite [14]. The tensile vibration of hydroxyl groups between the layers is responsible for the broad absorption peak observed at 3444 cm^−1^. The bending and bending vibrations of CO_3_^2−^ are represented by the absorption peaks at 1365 cm^−1^ and 670 cm^−1^, respectively. The absorption peaks at 3444, 1200, and 793 cm^−1^ are C−H stretching vibration bands. The tensile vibration of M−OH is shown by the absorption peak at 451 cm^−1^ (M stands for divalent cations). Following this, there was a little shift in the Zn peaks. This could be because Zn has a bigger ionic radius than Mg, which lengthens the M−OH bond.

The thermogravimetric curves of coal, raw coal, and the mixture of MgAl-LDHs are displayed in Figure 1e. The mass loss of MgAl-LDHs is comparatively high when compared to raw coal, as the figure illustrates. This could be because MgAl-LDHs in the coal-MgAl-LDHs mixture are more prone to thermal degradation at lower temperatures, which causes the mass loss of mixed samples to increase at low temperatures. As a result, adding MgAl-LDHs can prevent coal from burning spontaneously.

### 3.2. The Inhibition Properties of Zn-Modified Hydrotalcite-like Compounds with Different Contents

**Figure 2:**
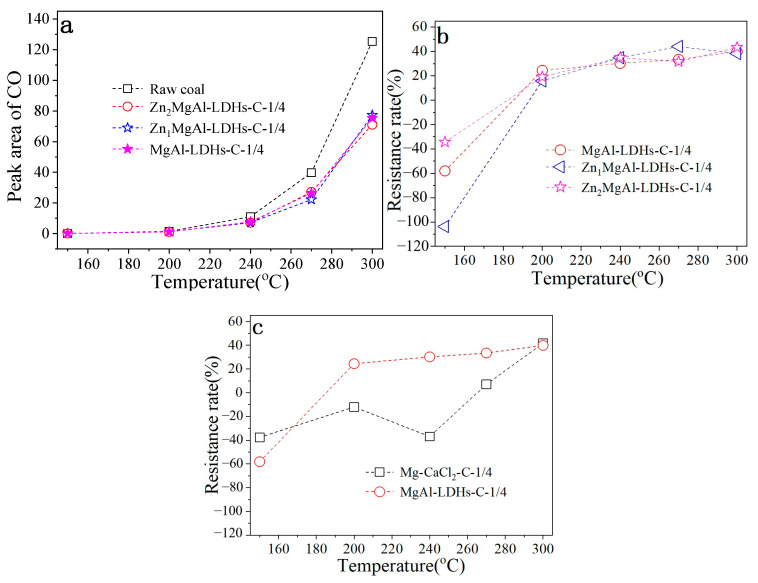
(**a**) The curve of CO emission from coal samples with different inhibitors versus temperature. (**b**) The inhibition properties of Zn-modified hydrotalcite. (**c**) Comparison of the inhibition performance between MgAl-LDHs and conventional commercial inhibitors.

Temperature-programmed oxidation was used to test the prepared hydrotalcite’s resistance. The curve of CO production with temperature increases when Zn-modified hydrotalcite is combined with new coal at a mass ratio of 1:4 is displayed in Figure 2a. Fresh coal samples were compared for CO production before and after the addition of hydrotalcite inhibitors. The results showed that the inhibitor significantly reduced CO production at the same temperature, demonstrating the hydrotalcite inhibitor’s clear flame retardancy. The amount of CO production reduces as the temperature rises, particularly in the high-temperature range of 240–300 °C, suggesting that the produced hydrotalcite inhibitor is highly temperature resistant. Furthermore, under the same circumstances, the variance in CO generation of Zn-modified hydrotalcite is not readily apparent. This could be because using hydrotalcite modified without zinc (MgAl-LDHs) can likewise produce higher flame retardancy while adding zinc to hydrotalcite coal has minimal effect on its ability to do so.

Temperature-programmed oxidation was used to test the prepared hydrotalcite’s resistance. The resistivity values of Zn-modified hydrotalcite combined with fresh coal at a 1:4 mass ratio are displayed in Figure 2b. The results demonstrate that the resistivity of hydrotalcite with varying Zn contents is negative at 150 °C. This could be because of the low CO content generated at low temperatures or because of the low temperature itself. Alternatively, it could be because the addition of hydrotalcite accelerated the breakdown of coal, producing more CO than the coal samples without the addition. Alcoa Hydrotalcite (MgAl-LDHs), Double the molar amount of Mg element Zn modified hydrotalcite (Zn_1_MgAl-LDHs), Twice the molar amount of Mg element Zn modified hydrotalcite (Zn_2_MgAl-LDHs) all have a flame-retardant effect on coal when the temperature is raised to 200 °C. The corresponding inhibition rates were 0.24, 0.16 and 0.19. Three types of hydrotalcite are more resistant to coal as the temperature rises. This demonstrates that hydrotalcite performs better at inhibiting coal in the 200−300 temperature range. Furthermore, at 300 °C, Zn_2_MgAl-LDHs had the strongest inhibitory effect, with an inhibition rate of 0.43. The resistance of MgAl-LDHs at the 200–300 °C temperature range is similar to that of the other two Zn-modified hydrotalcs (the resistance rate reaches 0.40 at 300 °C), which does not significantly indicate the significance of Zn modification when the preparation cost is taken into account. Consequently, it makes sense to select MgAl-LDHs as the retarding agent in further research.

The flame retardance performance of the MgAl-LDHs was compared with that of the commercial retardation agent (mixed preparation of CaCl_2_ and MgCl_2_, represented by Mg-CaCl_2_) currently used in Qiyi Coal Mine in order to further investigate the retardation performance of the prepared hydrotalcite inhibitor. Figure 2c illustrates that, in the same experimental conditions, mechanical mixing of pulverized coal and Mg-CaCl_2_ does not exhibit any flame-retardant effect in the 150–240 °C temperature range. It is only at 270 °C that the mixture starts to exhibit some flame-retardant effect, with a resistance rate of 0.07. The resistance rate increases quickly at 300 °C and reaches a resistance rate of 0.42, which is comparable to that of MgAl-LDHs. It can be observed that the synthesized MgAl-LDHs flame retardants demonstrated a better inhibitory effect when compared to commercial Mg-CaCl_2_ flame retardants. Furthermore, a review of the literature revealed that Mg-CaCl_2_ flame retardants only work through their hygroscope, which keeps the coal’s surface wet all the time to stop coal oxidation and heating [15,16]. Because of its overly basic resistance mechanism, it performs poorly as a flame retardant.

### 3.3. Spontaneous Combustion Tendency Test

The test coal sample was Qiyi Coal Industry’s No. 9 coal seam coal. Following crushing and screening, a coal sample with particle sizes between 0.18–0.25 mm was chosen, and it was vacuum-dried for 24 h at 40 °C. Testing was done using the coal spontaneous combustion tendency detecting platform. First, an asbestos layer was pre−applied to the insulated coal sample tank to prevent coal powder from obstructing the gas passage. After that, the device’s airtightness was examined, and the tank was filled with 30 min worth of nitrogen at a rate of 60 mL·min^−1^. The sample is programmed to heat up at a rate of 0.5 °C·min^−1^, the air is injected, the gas flow is set to 40 mL·min^−1^, the programmed heating chamber’s beginning temperature is set to 30 °C, and the termination temperature is set to 200 °C. Every 2 min, note the temperature of the coal sample and the furnace wall. When the desired temperature is reached, the test is terminated.

**Figure 3:**
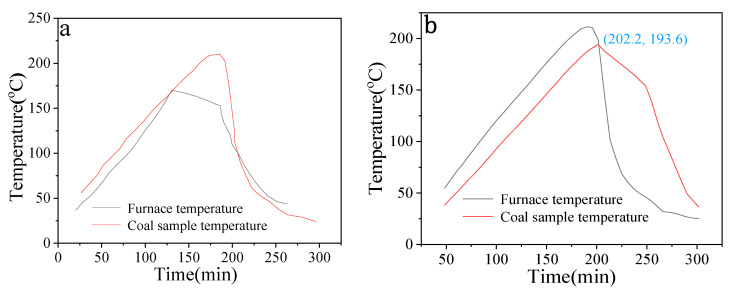
(**a**) Spontaneous combustion tendency of No. 9 coal seam in Qiyi coal industry. (**b**) Spontaneous combustion tendency of MgAl-LDHs and mechanically mixed coal samples.

The test results for the coal sample’s spontaneous combustion tendency are displayed in Figure 3a. The temperature of the coal sample climbs in tandem with the furnace’s temperature, reaching a crossover point at 146.2 °C. After that, the coal sample’s temperature keeps rising above that of the furnace, indicating that spontaneous combustion takes place in the coal sample and that an exothermic process starts at 146.2 °C following continuous air intake.

MgAl-LDHs were mechanically mixed with the coal sample at a certain mass ratio (1:4), in contrast to the MgAl-LDHs blocking agent. The temperature was raised under the same circumstances, and Figure 3b displays the test findings. The coal sample’s temperature rose in tandem with the furnace’s temperature, reaching a crossover point at 193.6 °C. It can be observed that MgAl-LDHs plays a flame retardant role by delaying the spontaneous combustion time and raising the temperature of spontaneous combustion after this temperature when compared to the coal sample without mixed with hydrotalcite inhibitor. In general, spontaneous combustion is more likely to occur in coal seams when the intersection temperatures range from 120 to 140 °C, and the ignition time is shorter than 90 days. The spontaneous combustion occurs in coal seams where the intersection temperature is between 140 to 160 °C, and the ignition period lasts longer than 90 days. It is difficult for a coal seam to spontaneously ignite when the temperature at the crossing point is higher than 160 °C. The No. 9 coal seam in Qiyi Coal Industry belongs to the spontaneous combustion coal seam category because its crossover temperature is approximately 146.2 °C. The cross−point temperature is approximately 193.6 °C when the inhibitor is added, and the coal seam’s tendency toward spontaneous combustion is difficult (the ignition period for spontaneous combustion is more than 180 days).

### 3.4. Study on Spontaneous Combustion Process of Coal

**Figure 4:**
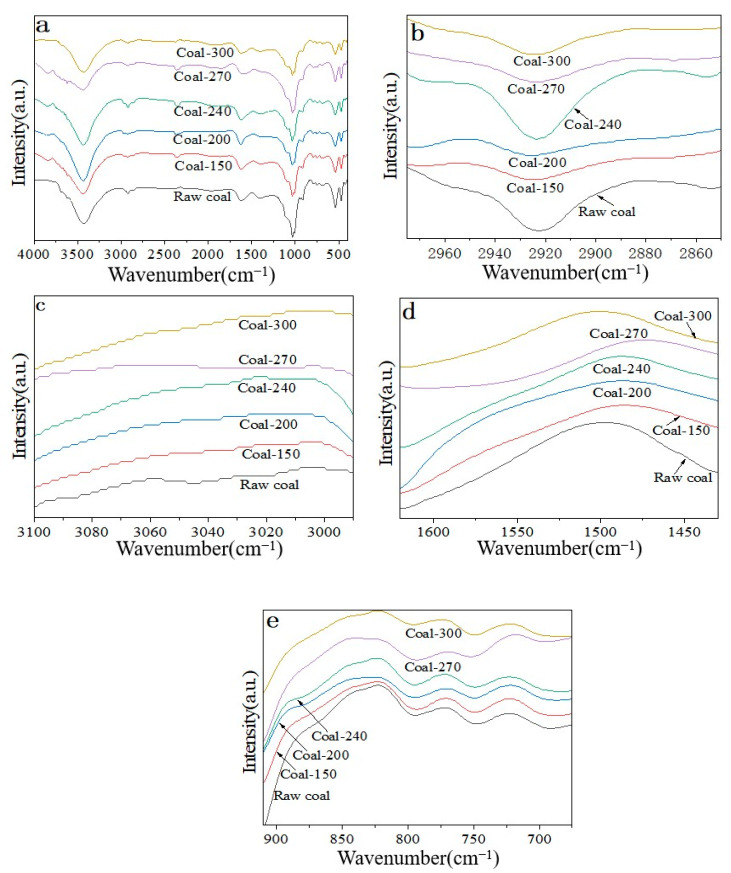
(**a**) Infrared spectra of fresh coal samples after oxidation at different temperatures. (**b**) Infrared spectra of aliphatic hydrocarbons (−CH_3_/−CH_2_−) after oxidation of fresh coal at different temperatures. (**c**) C−H infrared spectra of aromatic hydrocarbons after oxidation of fresh coal samples at different temperatures. (**d**) Infrared spectra of aromatic ring C=C after oxidation of fresh coal at different temperatures. (**e**) Infrared spectra of benzene substituted by fresh coal after oxidation at different temperatures.

After crushing and screening, coal samples from the No. 9 coal seam of Qiyi Coal Industry were chosen for this study. Coal samples with a particle size of 100–200 mesh were chosen for a temperature oxidation experiment that was scheduled, and the changes in the surface functional groups of the coal samples following oxidation at various temperatures were examined (Figure 4a). The infrared spectrum indicates that the primary functional groups found in coal are oxygen-containing functional groups (−OH, C=O, C−O, −COO−), aliphatic hydrocarbons (−CH_3_, −CH_2_−), and aromatic hydrocarbons (C−H, C=C, substituted benzene) [17]. Peak locations are listed in Table S1 of Supplementary Materials.

#### 3.4.1. Changes of Aliphatic Hydrocarbons

The spectral peak position of aliphatic hydrocarbon (−CH_3_/−CH_2_−) is primarily between 2975–2915 cm^−1^ and 2880–2850 cm^−1^, as shown in Table S1 of Supplementary Materials. Figure 4b illustrates how the amount of aliphatic hydrocarbon (−CH_3_/−CH_2_−) on the coal sample’s surface drops, grows, and then declines as the oxidation temperature rises. The findings suggest that at low temperatures, aliphatic hydrocarbons (−CH_3_/−CH_2_−) on the surface of coal samples are easily broken down by thermal oxidation. At approximately 240 °C, the content of aliphatic hydrocarbons (−CH_3_/−CH_2_−) increases, which could be the result of some new aliphatic hydrocarbon (−CH_3_/−CH_2_−) groups evolving from other groups as a result of coal sample oxidation. The freshly generated aliphatic hydrocarbon (−CH_3_/−CH_2_−) then undergoes another round of oxidation−based breakdown as a result of additional heat.

#### 3.4.2. Aromatic Hydrocarbon Change Law

C−H variation of aromatic hydrocarbons: The major range of the aromatic C−H stretching vibration is 3100–3000 cm^−1^. The peak intensity of aromatics C−H in the coal sample does not change significantly with temperature during the oxidation process, as shown by the infrared spectrum test results (Figure 4c). This suggests that the low content of aromatics C−H in the coal sample and its non−dominant oxidation may be the cause of this.

Change of aromatic ring C=C: The primary C=C expansion vibration of an aromatic ring is 1620–1430 cm^−1^. As demonstrated by the Fourier transform infrared spectrum test results (Figure 4d), a new aromatic ring C=C is generated on the surface of coal samples during the low-temperature oxidation process, while aromatic ring C=C is further oxidized and decreases with the increase of oxidation temperature. The infrared spectrum signal of aromatic ring C=C of fresh coal samples oxidized at different temperatures first increases and then decreases with the increase of oxidation temperature. Furthermore, there appears to be a clear shift in the aromatic ring C=C is an infrared spectrum signal peak, indicating that it has changed into another group.

Changes of substituted benzene: The major range of substituted benzene’s out-of-plane bending and stretching vibration is 910–675 cm^−1^. The spectral peak intensity changes of substituted benzene in aromatic hydrocarbons during oxidation at various temperatures in fresh coal samples are displayed in Figure 4e. According to the findings, the content of substituted benzene marginally dropped as the temperature rose. Only at high temperatures (270–300 °C) does substituted benzene content alter to some degree; at low temperatures (150–240 °C), substituted benzene as a whole does not change considerably with temperature. This demonstrates the strong temperature resistance of the substituted benzene on the coal sample’s surface, and the change in its content suggests that the coal sample is spontaneously burning at a higher temperature.

#### 3.4.3. Changes in Oxygen-Containing Functional Groups

**Figure 5:**
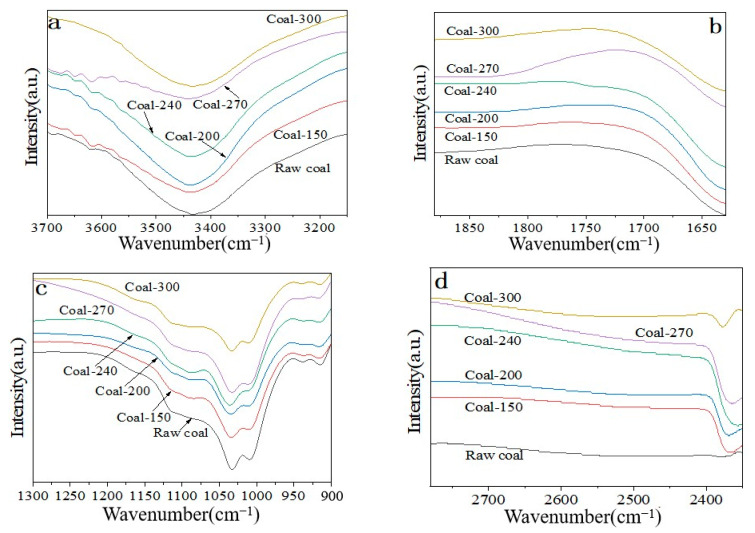
(**a**) Infrared Spectrum of −OH in oxygen-containing functional groups of fresh coal after oxidation at different temperatures. (**b**) C=O infrared spectra of oxygen-containing functional groups in fresh coal samples after oxidation at different temperatures. (**c**) C−O FTIR spectra of oxygen-containing functional groups in fresh coal samples after oxidation at different temperatures. (**d**) −COO− FTIR spectra of oxygen-containing functional groups in fresh coal samples after oxidation at different temperatures.

−OH change: In functional groups containing oxygen, the spectral peaks of −OH are primarily located between 3700–3625 cm^−1^ and 3550–3200 cm^−1^. The spectral peak signal of −OH in the oxygen-containing functional group on the coal sample’s surface progressively diminishes with increasing temperature, as seen in Figure 5a. The entire oxidation process of the coal sample occurs concurrently with the oxidative breakdown of the −OH group.

C=O change: In the oxygen-containing functional group, the stretching vibration of C=O is primarily seen between 1880–1785 cm^−1^ and between 1780–1630 cm^−1^. The infrared spectra of C=O in oxygen-containing functional groups of freshly oxidized coal samples at varying temperatures are displayed in Figure 5b. As the temperature rises, the signal strength of C=O in the oxygen-containing functional group gets stronger. The signal strength reaches its maximum at 270 °C, indicating that at that temperature, some groups oxidized to C=O, enhancing the C=O signal. It is evident that the signal strength of C=O in the oxygen-containing functional group hardly changes in the low-temperature stage (150–240 °C). The C=O signal decreased as the oxidation temperature rose to 300 °C, suggesting that the C=O group was progressively broken down by oxidation as the oxidation process advanced.

C−O change: The primary range of the C−O stretching vibration peak in a C−O bond is 1300–900 cm^−1^. The change in the C−O bond’s spectral peak strength in fresh coal samples during oxidation at various temperatures is depicted in Figure 5c. The peak intensity of the carbon-oxygen bond’s C−O spectrum on the coal sample’s surface is found to progressively decrease as the oxidation temperature rises, and this process is accompanied by a range of temperature values.

−COO− change: The principal stretching vibrational frequency of −COO− is 2780–2350 cm^−1^. The −COO− group increases during low-temperature oxidation treatment (150–240 °C), which may be caused by the oxidation of other groups on the coal surface into −COO− groups, according to the in situ Fourier transform infrared spectroscopy test findings (Figure 5d), increases the signal’s strength. The −COO− group undergoes more oxidation and breakdown as the oxidation temperature rises, significantly reducing the potency of its signal.

#### 3.4.4. Analysis of Coal Spontaneous Combustion Process

In conjunction with the outcomes of the experiment, the coal’s spontaneous combustion process can be summed up as follows: Major groups in oxygen-containing functional groups on the surface of the coal sample, such as aliphatic hydrocarbon (−CH_3_/−CH_2_−), aromatic ring C=C, −OH, C−O, and −COO− groups, slowly oxidize and continually release heat at low temperatures (below 240 °C). In the coal pile, heat builds up continuously, and the rate at which the coal sample oxidizes increases at a specific temperature (over 240 °C). On the surface of coal samples, groups like the oxygen-containing functional group C=O and aromatic ring C=C are created, and these groups steadily build up as temperature rises. After a particular temperature is reached, the newly created groups will undergo additional oxidation and breakdown, resulting in the release of energy [18]. Moreover, the surface of the coal sample exhibits substituted benzene oxidation at temperatures between 270–300 °C.

### 3.5. Study on Inhibition Mechanism

After crushing and screening a 100–200 mesh coal sample with a mesh diameter of No. 9 coal seam sample from Qiyi Coal Industry, the coal was mixed with various Zn elements to modify the hydrotalcite of magnesium and aluminum. The surface group changes at various temperatures were then examined, and the resistance mechanism of the prepared hydrotalcite was finally investigated.

**Figure 6:**
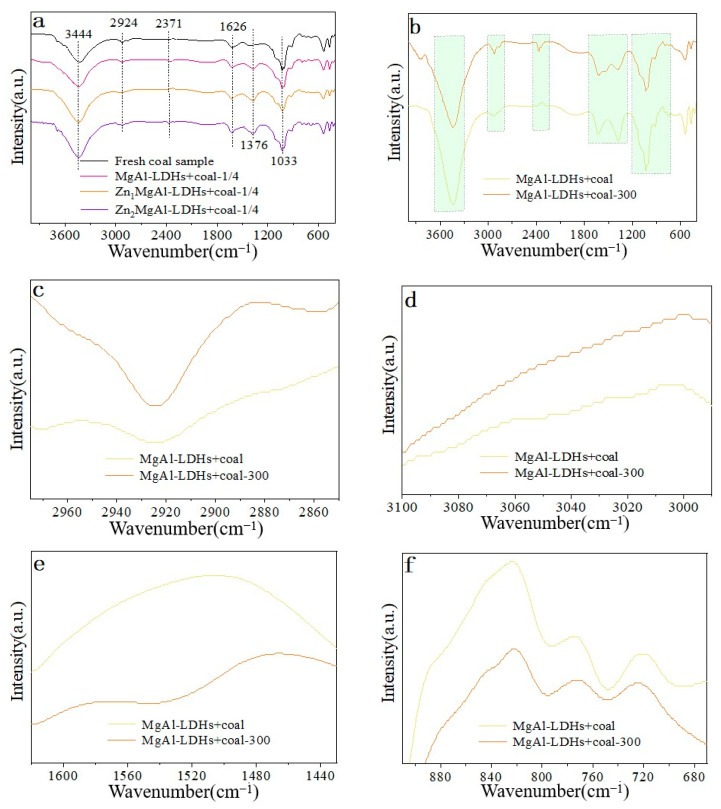
(**a**) FTIR spectra of fresh coal samples mechanically mixed with hydrotalcite-like compounds with different Zn contents. (**b**) Infrared spectra of hydrotalcite-like compounds after mechanical mixing with fresh coal samples before and after 300 °C treatment. (**c**) FTIR spectra of aliphatic hydrocarbons before and after 300 °C mechanical mixing of hydrotalcite-like compounds with fresh coal samples. (**d**) Infrared spectra of aromatic hydrocarbons after mechanical mixing of hydrotalcite-like compounds with fresh coal samples at 300 °C. (**e**) Infrared spectra of aromatic ring C=C after mechanical mixing of hydrotalcite-like compounds with fresh coal samples at 300 °C. (**f**) FTIR spectra of substituted benzene at 300 °C after mechanical mixing of hydrotalcite-like compounds with fresh coal samples.

Following mechanical mixing with new coal samples, the FTIR spectra of hydrotalcite with varying Zn levels in the 400–4000 cm^−1^ range are displayed in Figure 6a. According to the main characteristics of the infrared spectrum of the coal sample, the spectral peak is assigned to Table S1 of Supplementary Materials. It can be seen that before mixing hydrotalcite inhibitors. There are antisymmetric stretching vibrations of −CH_2_ (2930–2880 cm^−1^), symmetric variable Angle vibrations of −CH_3_ (1376 cm^−1^), out−of−plane bending vibrations of substituted benzene C−H (910–675 cm^−1^), phenol, alcohol, carboxylic acid −OH, or intermolecular association hydrogen bonds (3550–3200 cm^−1^) on the coal surface, aldehydes, ketones, carboxylic acids, esters, quinones C=O stretching vibration (1626 cm^−1^), and −OH stretching vibration of −COOH (2371 cm^−1^) [19,20]. The above functional groups are still present after adding hydrotalcite inhibitors with varying Zn contents, as can be observed, and the intensity of the peak (3444 cm^−1^) corresponding to water molecules increases. This could be because water molecules are present between hydrotalcite layers, which enhances the −OH signal.

Hydrotaltale-like coal samples were mechanically mixed with fresh coal samples and treated at 300 °C, and the changes in surface functional groups were compared before and after treatment. The alterations in surface functional groups were then examined. See Figure 6b for specifics. The figure’s shaded area evidently displays alterations in functional groups, a sign that the coal samples’ surface functional groups have undergone modification following treatment.

#### 3.5.1. Changes of Aliphatic Hydrocarbons

The spectral peak position of aliphatic hydrocarbon (−CH_3_/−CH_2_−) is primarily between 2975–2915 cm^−1^ and 2880–2850 cm^−1^, as Table S1 of Supplementary Materials. Figure 6c compares the change in peak intensity of −CH_3_/−CH_2_− in the coal samples oxidation process following the addition of MgAl-LDHs with temperature. The treated coal samples exhibited an increasing trend in the aliphatic hydrocarbon signal when compared to the non-oxidized coal samples. This might be because, as the temperature rises, the coal’s molecular structure produces a lot of −CH_3_/−CH_2_− during the sample’s thermal breakdown and oxidation process, which is followed by burning. The flame retardant affects the newly formed −CH_3_/−CH_2_−, which does not oxidize above about 300 °C. A significant amount of buildup causes the absorption peak to be enhanced.

#### 3.5.2. Aromatic Hydrocarbon Change Law

C−H variation of aromatic hydrocarbons: The major range of the aromatic C−H stretching vibration is 3100–3000 cm^−1^. The peak strength of aromatics C−H in the coal sample does not change significantly with temperature during the oxidation process after the addition of MgAl-LDHs, according to the Fourier transform infrared spectrum test results (Figure 6d). This suggests that the addition of an inhibitor effectively prevents the oxidation of aromatics C−H in the coal sample.

Change of aromatic ring C=C: The primary C=C expansion vibration of an aromatic ring is 1620–1430 cm^−1^. The findings of the Fourier transform infrared spectroscopy test (Figure 6e) show that, following the addition of MgAl-LDHs, the peak strength of the aromatic ring C=C in the coal sample diminishes as the temperature rises during the oxidation process. It demonstrates that some aromatic ring C=C is oxidized at roughly 300 °C, that the presence of an inhibitor inhibits the oxidation of aromatic ring C=C, and that a tiny quantity of aromatic ring C=C still exhibits an absorption signal.

Changes of substituted benzene: Substituted benzene’s out−of−plane bending and stretching vibration primarily falls between 910–675 cm^−1^. After hydrotalcite and fresh coal samples are mechanically mixed, the spectral peak intensity variations of substituted benzene in aromatic hydrocarbons are shown in Figure 5b both before and after 300 °C treatment (Figure 6f). While substituted benzene still exhibits a strong absorption signal, suggesting that the presence of an inhibitor prevents further oxidation and decomposition of substituted benzene, the results show that the peak strength of substituted benzene in the coal sample decreases with increasing temperature following the addition of MgAl-LDHs. This suggests that some substituted benzene is decomposed by oxidation at about 300 °C.

#### 3.5.3. Changes in Oxygen-Containing Functional Groups

**Figure 7:**
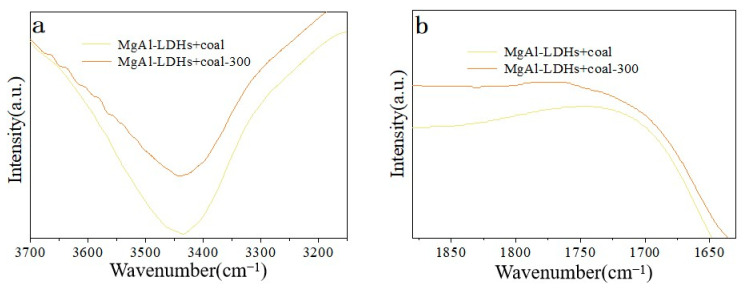

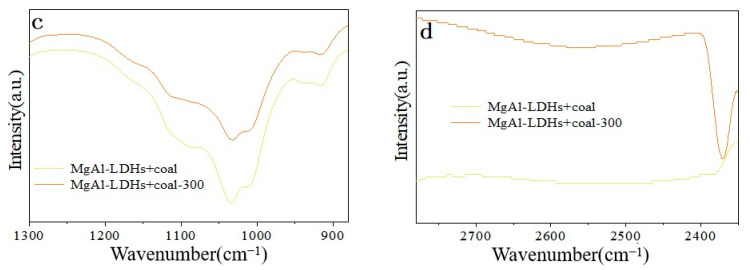
(**a**) After mechanical mixing of hydrotalcite-like compounds with fresh coal samples before and after 300 °C treatment −OH infrared spectra. (**b**) C=O FTIR spectra of hydrotalcite-like compounds mixed mechanically with fresh coal samples before and after 300 °C treatment. (**c**) C−O FTIR spectra of hydrotalcite-like compounds mixed mechanically with fresh coal samples before and after 300 °C treatment. (**d**) After mechanical mixing of hydrotalcite-like compounds with fresh coal samples before and after 300 °C treatment −COO− FTIR spectra.

−OH change: In functional groups containing oxygen, the spectral peaks of −OH are primarily located between 3700–3625 cm^−1^ and 3550–3200 cm^−1^. Figure 7a shows that although the peak of hydroxyl group (−OH) does not entirely vanish, the peak strength of −OH in coal samples diminishes with increasing temperature. This could be because, with the addition of the inhibitor, the −OH in the coal sample is shielded by hydrotalcite, allowing the −OH to be conserved. The −OH in the hydrotalcite layered structure may be the primary source of the reduced −OH peak signal.

C=O change: The principal positions of the C=O stretching vibration are 1880–1785 cm^−1^ and 1780–1630 cm^−1^. Figure 7b illustrates how the peak intensity of C=O in the coal sample changes with temperature following the addition of MgAl-LDHs. The peak strength of C=O in the coal sample following the addition of Zn_1_MgAl-LDHs does not change significantly with temperature during the oxidation process, as shown by the results of the Fourier transform infrared spectroscopy test. This suggests that the addition of the retarding agent effectively prevents the decomposition of C=O aromatics in the coal sample.

C−O change: The carbon-oxygen bond’s stretching vibration peak primarily lies between 1300–900 cm^−1^. The carbon-oxygen bond’s C−O spectral peak intensity changes before and after 300 °C treatment following the mechanical mixing of hydrotalc and fresh coal sample, as shown in Figure 7c. The findings demonstrate that, following the addition of MgAl-LDHs, the peak strength of the C−O bond in the coal sample decreases with increasing temperature, suggesting that some substituted benzene is oxidized and decomposed at about 300 °C. However, the C−O bond still exhibits a strong absorption signal, suggesting that the inhibitor’s presence prevents the C−O bond from further oxidizing and decomposing.

−COO− Change: The principal stretching vibrational frequency of −COO− is 2780–2350 cm^−1^. The −COO− group increases during oxidation treatment, according to the in−situ Fourier transform infrared spectroscopy test findings (Figure 7d). This could be because additional groups on the coal surface oxidize to become −COO− groups, which amplifies the signal strength.

The oxidation of organic groups on the surface of coal is significantly inhibited after the addition of MgAl-LDHs, indicating that the prepared MgAl-LDHs inhibitor has strong flame-retardant properties of coal, based on changes of different functional groups on the surface of coal samples before and after oxidation treatment.

#### 3.5.4. Inhibition Mechanism Analysis

The inhibitory mechanism of hydrotalc−like LDHs on coal spontaneous combustion was investigated, in conjunction with the experimental data presented in this study, and the impact of LDHs on functional groups in the oxidation process. Thus, a theoretical coupling mechanism between the heat effect and adsorption between LDHs and coal is presented. Here is a description of the mechanism: LDHs cover the surface of coal and adsorb, blocking the contact between oxygen and coal and preventing oxygen from interacting with coal on the surface of coal because of the presence of bound water and adsorbed anions between layers of LDHs. In order to prevent the more active −COO− oxygen-containing functional groups in coal from completing the low-temperature oxidation reaction, oxygen-containing functional groups, such as −COO− in coal, can establish a weak hydrogen bond with −Oh on LDHs. In addition, the endothermic breakdown of LDHs has the ability to absorb heat released during the low-temperature oxidation stage of coal and produce CO_2_, H_2_O, and other gases. This slows down the rate at which coal temperature rises and may therefore contribute to the prevention of coal spontaneous combustion.

#### 3.5.5. Hydrotalc-like Coal Spontaneous Combustion Resistance Technology

This work pioneered the technology of employing MgAl-LDHs as a flame-retardant material to retard the spontaneous burning of coal. MgAl-LDHs have high flame retardancy against coal spontaneous combustion, according to earlier tests. However, a specific amount of CaCl_2_ (one-fourth the mass of the created MgAl-LDHs inhibitor) is added to the MgAl-LDHs suspension in order to further increase the film-forming property of the MgAl-LDHs inhibitor on the coal surface [21]. The primary purpose of adding CaCl_2_ is to enhance flame-retardant performance by equally coating the surface of the coal particles with the migAL−LDHS suspended particles thanks to their water absorption properties and viscosity.

## 4. Conclusions

In this paper, by using the coprecipitation method, Zn-modified Mg-Al hydrotalcite was created, and its coal resistance was investigated. The findings indicate that the Zn level of Mg-Al hydrotalcite has minimal impact on its flame retardancy. Therefore, in order to prevent coal from spontaneously combusting in drift, Mg-Al hydrotalcite (MgAl-LDHs) devoid of the Zn element was chosen as the flame-retardant material. The spontaneous combustion propensity experiment demonstrates that adding MgAl-LDHs to coal seam No. 9 in Qiyi coal industry can raise the temperature of the exothermic reaction from 146.2 °C to 193.6 °C. Thus, the spontaneous combustion period of the coal in the No. 9 coal seam of Qiyi coal industry is extended from 90 days to 180 days, which can play a role in temporary fire prevention and extinguishing in the excavation roadway. Coating and adsorbing on the coal surface, LDHs stop oxygen from reacting on the coal surface by obstructing the interface between coal and oxygen. The more active −COO− oxygen functional groups in coal could not be oxidized at low temperatures because the −COO− and other oxygen-containing functional groups in coal can create weak hydrogen bonds with −OH on LDHs. In addition, LDHs have the ability to absorb heat from the low-temperature oxidation of coal and produce CO_2_, H_2_O, and other gases. This slows down the rate at which coal temperatures rise and, in part, acts as a brake on coal’s spontaneous combustion. The method used in this work to retard coal spontaneous combustion used magnesium alkali metal hydroxides (MgAl-LDHs) as a flame retardant. Adding CaCl_2_ to the suspension of magnesium−lead hydroxide (MgAl-LDH) particles strengthens their uniform coverage on the surface of coal particles, which is advantageous for consolidating their flame retardancy. This is based on the water absorption and viscosity of CaCl_2_. However, the strength of the subsequent shotcrete is unaffected by the flame-retardant film that forms after the shotcrete cover.

## Data Availability

The original contributions presented in the study are included in the article/Supplementary Material, further inquiries can be directed to the corresponding author.

## Supplementary Materials

The following supporting information can be downloaded at: https://www.mdpi.com/article/10.3390/ma18010070/s1. Figure S1: The collection and preparation process of coal samples; Table S1: Attribution table of main characteristic peaks of infrared spectra of coal samples.
