# Supplementary material for: Preparation and Flame-Retardant Mechanism of MgAlZn-Based Hydrotalcite-like Coal Spontaneous Combustion Inhibitor
Source: Materials (Basel). 2024 Dec 27;18(1):70. doi: 10.3390/ma18010070 (PMC11721735; doi:10.3390/ma18010070)
Supplement: Supplementary file 1 [file materials-18-00070-s001.zip › materials-3009004-supplementary.pdf]

materials

Supplementary Materials

Preparation and Flame–Retardant Mechanism of MgAlZn–Based Hydrotalcite–like Coal Spontaneous Combustion Inhibitor

Lei Li, Yaohui Li, Zulin Li, Lingling Wu, Jingchuan Gou, Xingrong He, Chenxi Xu, Caijing Xie and Wanyue Wu

The samples were dried by V70 Fourier transform infrared spectrometer (FTIR) made by Brucker Company, then mixed with dried potassium bromide, ground and pressed. The range of measurement is 4000 - 400cm^{-1}, the resolution is 4 cm^{-1}, scanning 32 times, using the deuterated triglycoside peptide detector. The S4800 cold field emission scanning electron microscope (SEM) produced by Hitachi company was used to observe the morphology of the samples. The resolution of secondary electronic image is 1.0 nm (15 kV) , with semiconductor backscatter probe, the sensitivity is 0.1 z, the acceleration voltage is 0.5-30 kV, 0.1 kV per step, and the magnification is ×20 - ×800000. In the experiment, a small amount of powder samples were coated on the conductive adhesive, and after spraying gold, they were directly fixed on the sample stage for SEM observation. A 50mm^2 SDD silicon drift crystal x-ray energy spectrum detector is configured to analyze elements in the range Be4 - U92 for Quantitative analysis samples. The thermo-gravimetric curves of hydrotalcite-like samples during the heating process were measured by STA449F3 synchronous thermal analyzer. Test conditions: temperature rising rate 10 ^{o}C/min, temperature measuring range 25 - 900 ^{o}C, air flow rate 20 mL/min, reference material Al_2O_3 solid powder.

Table S1. Attribution table of main characteristic peaks of infrared spectra of coal samples.

Peak type		Spectral peak number	Peak position /cm^{-1}	The functional group	Peak assignment
**Aliphatic hydrocarbons**	-CH_3	1	2975-2945	-CH_3	the antisymmetric telescopic vibration of -CH_3
		2	2930-2880	-CH_2	the antisymmetric telescopic vibration of -CH_2
		3	2875±5	-CH_3	the symmetric telescopic vibration of -CH_3
	-CH_2	4	2855±5	-CH_2	the symmetric telescopic vibration of -CH_2
		5	1470±5	-CH_2	-CH_2 angular vibration
		6	1460±5	-CH_3	-CH_3 antisymmetric angular vibration, characteristic frequency
		7	1375±5	-CH_3	-CH_3 symmetric angular vibration
Aromatic hydrocarbons	Aromatic hydrocarbons	8	3100-3000	-CH	C-H telescopic vibration of aromatic hydrocarbons
		9	1910-1900	C-C/C-H	C-C, C-H vibrational frequency doubling and closing peaks of benzene
	Aromatic rings	10	620-1430	C=C	C = C skeleton stretching vibration in aromatic ring/dense ring
	Substituted benzene	11	910-675	C-H	C-H out of plane bending vibration of substituted benzenes
Oxygen-containing functional groups	-OH	12	3700-3625	-OH	Free -OH bond, judge alcohol, phenol, organic acids
		13	3624-3610	-OH	-OH self-association hydrogen bond, ether o and -OH hydrogen bond
		14	3550-3200	-OH	Phenol, alcohol, carboxylic acid -OH or intermolecular hydrogen bond
		15	1500-1350	-OH	- OH in-plane bending vibration
	C=O	16	1880-1785	C=O	C=O stretching vibration
		17	1780-1630	C=O	C=O stretching vibration of aldehyde, ketone, carboxylic acid, ester, quinone
	C-O	18	1330-900	C-O	Phenol, alcohol, ether, Ester carbon-oxygen bond
	-COO-	19	2780-2350	-COOH	-COOH's -OH telescopic vibration

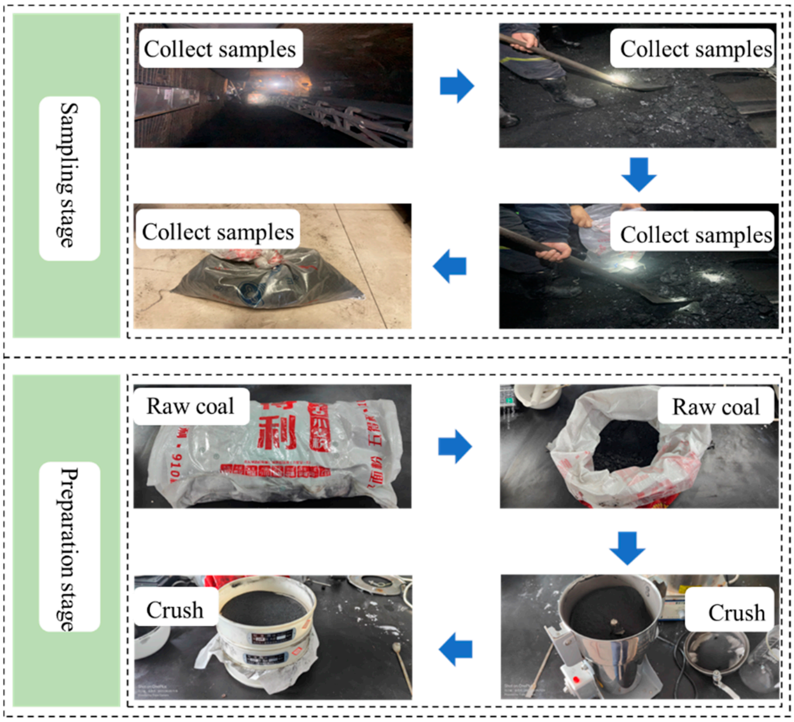

Figure S1. The collection and preparation process of coal samples.
